# LC-MS/MS-QTOF dataset of compounds detected in kelulut honey of the stingless bees, *Heterotrigona* itama and *Tetrigona binghami* from Kuantan, Pahang, Malaysia

**DOI:** 10.1016/j.dib.2023.109409

**Published:** 2023-07-12

**Authors:** Che Muhammad Khairul Hisyam Ismail, Nicholas M.H. Khong, Azmir Ahmad, Khairani Idah Mokhtar, Widya Lestari, Basma Ezzat Mustafa Alahmad, Azzmer Azzar Abdul Hamid, Mohd Ridzuan Mohd Abd Razak, Azlini Ismail

**Affiliations:** a Department of Biotechnology, Kulliyyah of Science, International Islamic University Malaysia, 25200 Kuantan, Pahang, Malaysia; b Institute of Planetary Survival for Sustainable Well-Being (PLANETIIUM), International Islamic University Malaysia, 25200 Kuantan, Pahang, Malaysia; c School of Pharmacy, Monash University Malaysia, Jalan Lagoon Selatan, 47500, Bandar Sunway, Selangor, Malaysia; d Department of Basic Medical Science for Nursing, Kulliyyah of Nursing, International Islamic University Malaysia, 25200 Kuantan, Pahang, Malaysia; e Department of Fundamental Dental and Medical Sciences, Kulliyyah of Dentistry, International Islamic University Malaysia, 25200 Kuantan, Pahang, Malaysia; f Herbal Medicine Research Centre, Institute for Medical Research, National Institutes of Health, 40170 Shah Alam, Selangor, Malaysia

**Keywords:** Honey, LC-MS/MS-QTOF, *Heteroitama trigona*, *Tetrigona binghami*, Stingless bee

## Abstract

Honey is a sustainable nutritious substance which has been incorporated into the human diet since ancient times for its health and remedial benefits. Stingless bee honey or kelulut honey (KH) is well-known in Malaysia and has received high demand in the market due to its distinctive unique flavour. Its composition, colour, and flavour are majorly affected by the geographical location, floral source, climate, as well as the bee species. This data article presents the nontargeted metabolite profiling of the extracts of KH of *Heterotrigona itama* and *Tetrigona binghami* bee species. The KH was collected from three nests in Kuantan, Pahang, which is situated in the east coast of Peninsular Malaysia. The extracts were prepared using sugaring-out assisted liquid-liquid extraction (SULLE) method and the Liquid Chromatography–Tandem Mass Spectrometry with Quadrupole Time-of-Flight, operated in the negative ion mode, was used to identify compounds in the extracts. The data processing revealed the presence of 35 known compounds in the KH1 extract by *Heterotrigona itama* collected from Bukit Kuin, 38 compounds in the KH2 extract by *H. itama* collected from Indera Mahkota, whilst 50 known compounds were present in KH3 extract by *Tetrigona binghami species* from Indera Mahkota. This data article contains the m/z values, retention times, and the METLIN database search hit identities of the compounds and their respective classes.

**Specifications Table**

**Table:** 

Subject	Chemistry
Specific subject area	Chemicals, Natural product research, Spectrometry
Type of data	Table, Figures
How the data were acquired	Data on the chemical compounds in the SULLE extracts of KH obtained from *Heterotrigona itama* and *Tetrigona binghami* bee species were acquired using Liquid Chromatography Tandem Mass Spectrometry Quadrupole-Time-of-Flight (Agilent 1290 Infinity LC system coupled to Agilent 6520 Accurate-Mass Q-TOF mass spectrometer with dual ESI source).
Data format	Raw and analyzed
Description of data collection	The KH samples were harvested from the nests of *Heterotrigona itama* and *Tetrigona binghami* bee species and stored in a 500 mL airtight glass bottle at 4°C until further extraction using SULLE approach [1].
Data source location	The KH samples from *Heterotrigona itama* was collected from two beehives at two different locations i.e. the Bukit Kuin forest (3°52′54.9′′N 103°12′27.4′′E) (designated as KH1), and the beehive located in front of Kuliyyah of Nursing, International Islamic University Malaysia (IIUM), Indera Mahkota, Kuantan (3°50′28.7′′N 103°18′05.8′′E) (designated as KH2). For the third sample of Kelulut Honey (designated as KH3) from *Tetrigona binghami* species, the beehive was located at Sultan Haji Ahmad Shah Mosque, IIUM Kuantan (3°50′21.6′′N 103°18′14.3′′E). All the samples were collected on 30^th^ October 2022.
Data accessibility	The complete dataset is accessible at the Mendeley Repository: http://dx.doi.org/10.17632/w9wtvf52vd.1[2]

## Value of the Data

• This dataset presented the chemical profiles of Kelulut honey of the stingless bees, *Heterotrigona itama* and *Tetrigona binghami*, collected from three locations in Kuantan region of the eastcoast Malaysia.
• As honey composition can be impacted by geographical locations and stingless bee species [3,4], this data provide information on the compositions of KH in this particular region which would greatly benefit the researchers of traditional and complementary medicine and natural products.
• This dataset would also be useful in postulating the potential bioactive compounds that may contribute to the numerous health benefits of KH. The health-promoting effects of KH have been linked to its biological properties such as its antioxidant, anti-inflammatory, anti-diabetic, anti-ageing, antimicrobial, anti-cancer, and hypolipidemic effects as demonstrated in many of the previous *in vitro* and *in vivo* studies [5,6]. All in all, these will aid in future identification of the KH potential biomarker with regard to any particular therapeutic applications.

## 1 Objective

Kelulut honey (KH) is a type of honey that derived from stingless bee with a distinctive unique flavour. KH is known to possess numerous biological activities such as anti-inflammatory, antioxidant, anti-diabetic, anti-ageing, antimicrobial, anti-cancer and also hypolipidemic effects [5,6]. There are approximately 500 stingless bee species which dwell in the warm and humid forests worlwide. KH composition and properties depend on the bee species, the floral sources and the nectar that the bees collected, storage conditions, environmental factors, processing methods and also the geographical origin; due to these factors, there were some variations found across KH samples from around the globe in terms of its composition [3,4]. As there are still a dearth of information regarding KH compositions from Malaysia, this study aimed to provide a dataset on the phytochemical compositions in KH which derived from two stingless bee species, *Heterotrigona itama* and *Tetrigona binghami*, collected from Kuantan region of the eastcoast Malaysia.

## 2 Data Description

**Fig. 1:**
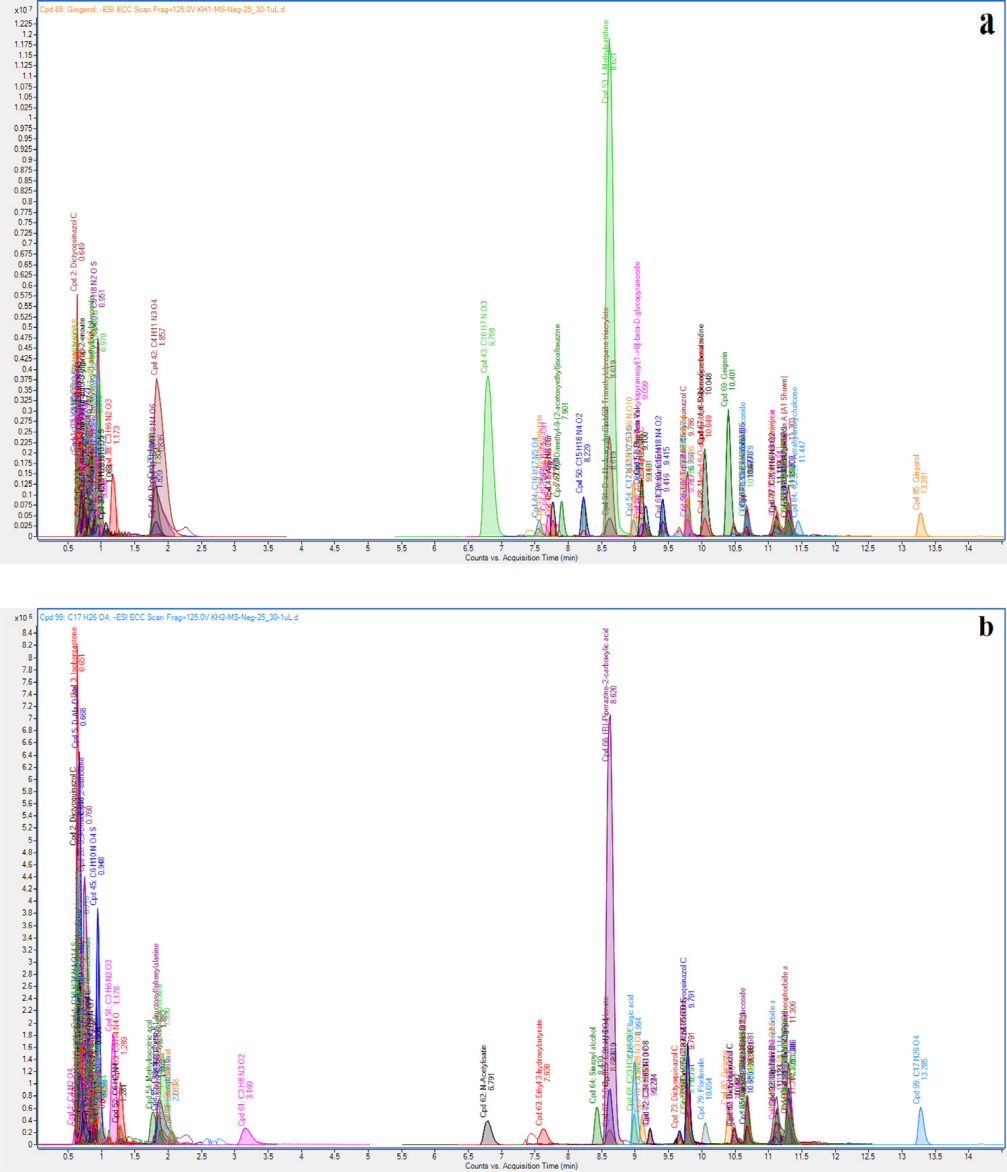

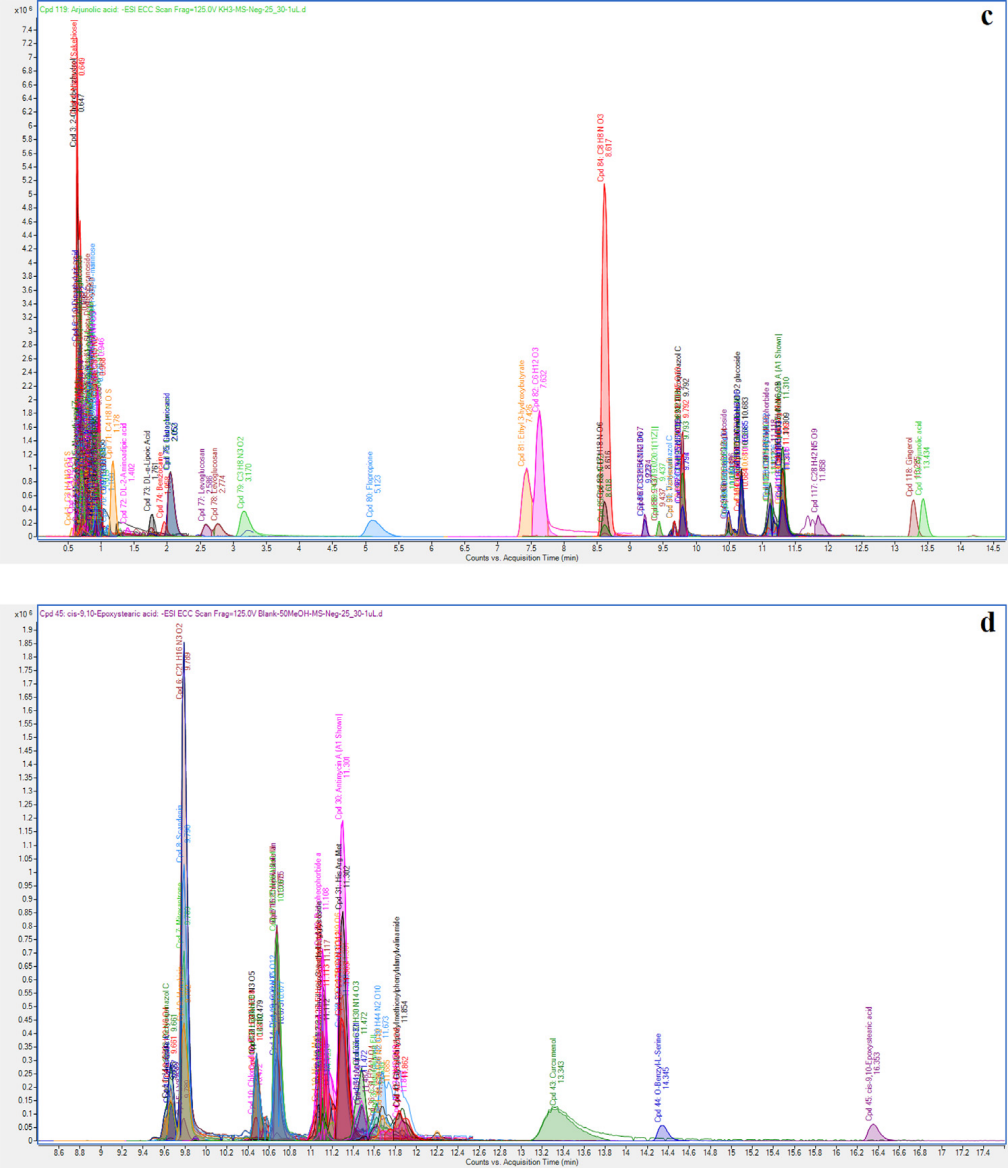
LC-MS/MS-QTOF chromatograms of KH extracts: (a) KH of *Heterotrigona itama* collected from Bukit Kuin (KH1); (b) KH of *Heterotrigona itama* from Indera Mahkota (KH2); (c) KH of *Tetrigona binghami* from Indera Mahkota (KH3); and (d) blank (carrier solvent).

Fig. 1 depicts the total ion chromatogram of kelulut honey samples (KH1, KH2 and KH3) obtained by analyzing the SULLE extracts using LC-MS/MS-QTOF. The raw dataset was further matched with the Agilent MassHunter METLIN Metabolomics Database and Library for the identification of the detected compounds. The list of the tentatively identified compounds in KH1, KH2, and KH3 extracts with their retention times (RT), molecular mass, molecular formula generation (MFG), MFG Diff (ppm), Database (DB) formula, DB Diff (ppm), and hits (DB) are presented in Tables 1, 2, and 3. The raw data of all the identified compounds from the KH samples can be found in the Mendeley data repository at this link: http://dx.doi.org/10.17632/w9wtvf52vd.1 [2]. After removing duplicate detection of similar compounds found in each extract, there were a total of 35 known compounds identified from KH1, followed by 38 known compounds identified from KH2, and 50 known compounds identified from KH3. Table 4 tabulates the variation in composition for a total of individual 110 compounds accross KH samples.

**Table 1:** The identified compounds from KH1 extract.

No	Name	MFG Formula	MFG Diff (ppm)	DB Formula	DB Diff (ppm)	Hits (DB)	Retention time (RT)	Mass	Mass-to-charge ratio (m/z)	Height	Width	Volume	Volume (%)
1	1,10-Phenanthroline			C12 H8 N2	4.44	1	0.659	180.0679	215.0373	2643724	0.086	14493344	2.56
2	Pyocyanin	C13 H10 N2 O	-1.91	C13 H10 N2 O	-1.91	2	0.715	210.0797	245.0491	283070	0.063	1121566	0.20
3	3-(3-Methylbutyl)tricetin 5-neohesperidoside			C32 H40 O16	-0.3	1	0.717	680.2318	679.2228	286036	0.068	1063881	0.19
4	5,8,11-Dodecatriynoic acid			C12 H12 O2	4.86	6	0.721	188.0828	223.0523	576051	0.052	1607446	0.28
5	Ulexone B	C25 H22 O5	-1.39	C25 H22 O5	-1.39	3	0.742	402.1473	401.1403	322045	0.134	2289329	0.40
6	ethyl 2-cyano-3-(1h-indol-3-yl)prop-2-enoate	C14 H12 N2 O2	2.68	C14 H12 N2 O2	2.68	4	0.777	240.0892	239.0823	897538	0.098	6651404	1.17
7	Zizybeoside II	C25 H38 O16	-3.06	C25 H38 O16	-3.06	1	0.836	594.2178	593.2107	412739	0.079	2683241	0.47
8	Lys Phe Cys			C18 H28 N4 O4 S	-2.09	6	0.844	396.184	431.1523	668800	0.078	3843960	0.68
9	Metominostrobin	C16 H16 N2 O3	-4.31	C16 H16 N2 O3	-4.31	10	0.845	284.1173	283.1102	758280	0.067	3550501	0.63
10	Dihydroxycarbazepine	C15 H14 N2 O3	-2.17	C15 H14 N2 O3	-2.18	2	0.854	270.101	269.0938	384925	0.089	2415592	0.43
11	(S)-Multifidol 2-[apiosyl-(1->6)-glucoside]	C22 H32 O13	0.82	C22 H32 O13	0.82	1	0.89	504.1839	503.1768	790399	0.075	3522779	0.62
12	Tyrosyl-Methionine	C14 H20 N2 O4 S	2.81	C14 H20 N2 O4 S	2.81	10	0.9	312.1135	311.1063	544421	0.066	2030881	0.36
13	Adipamide			C6 H12 N2 O2	-2.39	2	0.904	144.0902	179.0594	486834	0.062	1933265	0.34
14	N-Desethylquinagolide glucuronide	C24 H37 N3 O9 S	1.05	C24 H37 N3 O9 S	1.05	1	0.973	543.2245	542.2176	285347	0.077	1512358	0.27
15	Valsartan			C24 H29 N5 O3	1.84	8	0.984	435.2262	470.1945	322241	0.05	1501181	0.26
16	L-Canaline	C4 H10 N2 O3	-2.55	C4 H10 N2 O3	-2.55	2	0.996	134.0695	133.0622	294344	0.059	1078694	0.19
17	Imetit			C6 H10 N4 S	1.82	1	1.804	170.0623	205.0315	1218781	0.106	8513882	1.50
18	Propane-1-thiol			C3 H8 S	-3.54	3	1.82	76.0349	111.0043	342113	0.113	2498771	0.44
19	Ethyl 3-hydroxybutyrate	C6 H12 O3	-3.09	C6 H12 O3	-3.1	10	7.631	132.0791	131.0718	282837	0.123	2415506	0.43
20	HoPhe-HoPhe-OH	C25 H24 N2 O6	2.32	C25 H24 N2 O6	2.32	7	7.702	448.1624	447.1552	434529	0.049	1889044	0.33
21	Arg His Thr			C16 H28 N8 O5	1.14	6	7.769	412.2178	447.1869	654177	0.052	3101213	0.55
22	6,7-Dimethyl-9-(2-acetoxyethyl)isoalloxazine	C16 H16 N4 O4	-4.34	C16 H16 N4 O4	-4.34	3	7.901	328.1186	327.1113	521014	0.066	4279265	0.76
23	D-α-Hydroxyglutaric acid	C5 H8 O5	-0.28	C5 H8 O5	-0.29	10	8.619	148.0372	147.03	398886	0.105	3123105	0.55
24	Trimethylolpropane triacrylate			C15 H20 O6	-1.25	8	8.619	296.1264	331.0957	1948530	0.091	14621877	2.58
25	1-Methylxanthine	C6 H6 N4 O2	-1.55	C6 H6 N4 O2	-1.55	4	8.621	166.0493	165.0422	10334374	0.118	90080304	15.90
26	1-Octen-3-ol-3-o-beta-D-xylopyranosyl(1->6)-beta-D-glucopyranoside	C19 H34 O10	-1.14	C19 H34 O10	-1.14	10	9.099	422.2157	421.2085	262173	0.067	1260232	0.22
27	Phe Asn Val	C18 H26 N4 O5	0.8	C18 H26 N4 O5	0.8	6	9.1	378.19	377.1828	1143245	0.069	5675099	1.00
28	Schizonepetoside C	C16 H26 O7	3.87	C16 H26 O7	3.87	10	9.16	330.1666	329.1593	278608	0.072	1567702	0.28
29	Proparacaine			C16 H26 N2 O3	3.86	2	9.416	294.1932	329.1625	301187	0.074	1726815	0.30
30	Gln Trp Glu			C21 H27 N5 O7	3.37	6	9.787	461.1895	496.1586	321231	0.088	2077477	0.37
31	4,4′-Stilbenedicarboxamidine	C16 H16 N4	-0.55	C16 H16 N4	-0.54	1	10.048	264.1376	263.1306	1153132	0.081	12451485	2.20
32	Methyl 6-O-digalloyl-beta-D-glucopyranoside	C17 H18 N4 O2	-0.26	C17 H18 N4 O2	-0.26	5	10.049	310.1431	309.1358	377127	0.087	2423220	0.43
33	Geigerin	C15 H20 O4	-1.87	C15 H20 O4	-1.87	10	10.401	264.1367	263.1294	1451364	0.076	19326424	3.41
34	Cinncassiol D2 glucoside			C26 H42 O11	-4.16	1	10.679	530.2749	565.2442	376541	0.096	2530142	0.45
35	4,2′,3′,4′-Tetrahydroxychalcone	C15 H12 O5	-1.08	C15 H12 O5	-1.08	10	11.447	272.0688	271.0616	327418	0.086	2063698	0.36

**Table 2:** The identified compounds from KH2 extract.

No	Name	MFG Formula	MFG Diff (ppm)	DB Formula	DB Diff (ppm)	Hits (DB)	Retention time (RT)	Mass	Mass-to-charge ratio (m/z)	Height	Width	Volume	Volume (%)
1	Isobergaptene			C12 H8 O4	-4.32	6	0.651	216.0432	215.0355	4382884	0.07	32108084	7.29
2	D-Ala-D-Ala			C6 H12 N2 O3	1.42	9	0.668	160.0846	195.0539	5882680	0.082	29458050	6.69
3	Fluticasone propionate			C25 H31 F3 O5 S	-4.92	1	0.678	500.1869	535.1563	395945	0.054	1284760	0.29
4	4-Methylene-L-glutamine			C6 H10 N2 O3	4.19	3	0.679	158.0685	193.0379	1089017	0.057	4537348	1.03
5	Theobromine	C7 H8 N4 O2	-4.7	C7 H8 N4 O2	-4.69	3	0.698	180.0656	179.0583	1549084	0.117	7988196	1.81
6	Strictosamide			C26 H30 N2 O8	14.84	6	0.712	498.1928	533.1688	274769	0.09	786309	0.18
7	5-Hydroxy-4-methoxy-3-methyl-2,6-canthinedione	C16 H12 N2 O4	4.31	C16 H12 N2 O4	4.31	9	0.717	296.0784	295.071	600081	0.068	1763956	0.40
8	L-Arginine phosphate			C6 H15 N4 O5 P	0.91	1	0.717	254.0778	253.0704	343201	0.05	1400235	0.32
9	2′-Deoxymugineic acid			C12 H20 N2 O7	-5.48	3	0.718	304.1287	339.0976	210386	0.049	553461	0.13
10	Epicatechin 5,3′-dimethyl ether			C17 H18 O6	1.63	9	0.728	318.1098	353.0792	404802	0.087	1691666	0.38
11	Val Asp			C9 H16 N2 O5	0.53	10	0.743	232.1058	267.0752	370979	0.073	1955375	0.44
12	1-Imidazolelactic acid			C6 H8 N2 O3	0.73	5	0.753	156.0534	191.0228	520704	0.089	2890078	0.66
13	3,3-Dimethyl-1,2-dithiolane	C5 H10 S2	-5.15	C5 H10 S2	-5.15	3	0.76	134.0231	133.0158	4201739	0.091	23796972	5.41
14	N6-Acetyl-N6-hydroxy-L-lysine			C8 H16 N2 O4	4.91	9	0.779	204.11	239.0796	883048	0.107	7111446	1.62
15	Fluticasone 17-carboxylic acid			C21 H26 F2 O5	-1.05	1	0.848	396.1752	431.1437	232593	0.082	1369581	0.31
16	6-Hydroxyl-1,6-dihydropurine ribonucleoside	C10 H14 N4 O5	-4.64	C10 H14 N4 O5	-4.64	6	0.857	270.0977	269.0904	456588	0.091	2756504	0.63
17	Arabinosylhypoxanthine	C10 H12 N4 O5	-4.01	C10 H12 N4 O5	-4.01	10	0.86	268.0818	267.0745	345745	0.066	1538640	0.35
18	Flumethasone			C22 H28 F2 O5	0.84	3	0.874	410.1901	445.1601	338678	0.065	1744198	0.40
19	Thr-Thr-OH			C14 H18 N2 O8	-4.93	1	0.892	342.108	377.0773	178594	0.048	474669	0.11
20	Neu5Acα2-6GalNAcα-Thr			C26 H43 N3 O17	-3.31	1	0.897	669.2615	704.2307	201218	0.031	547091	0.12
21	L-Canaline	C4 H10 N2 O3	3.13	C4 H10 N2 O3	3.13	2	0.998	134.0687	133.0614	335451	0.054	1117381	0.25
22	Methylisocitric acid	C7 H10 O7	-5.86	C7 H10 O7	-5.86	5	1.783	206.0439	205.0363	496027	0.134	4198428	0.95
23	Coproporphyrin II	C36 H38 N4 O8	4.92	C36 H38 N4 O8	4.92	5	1.862	654.2657	653.2589	357373	0.12	2890782	0.66
24	N-(1-Deoxy-1-fructosyl)phenylalanine	C15 H21 N O7	-4.01	C15 H21 N O7	-4.01	1	1.882	327.1331	326.126	636147	0.176	7344778	1.67
25	Isopropyl apiosylglucoside			C14 H26 O10	1.22	1	1.916	354.1522	389.1215	215459	0.167	2403905	0.55
26	Benzocaine	C9 H11 N O2	-0.44	C9 H11 N O2	-0.43	10	1.93	165.0791	164.0718	1293444	0.136	11981000	2.72
27	Levoglucosan	C6 H10 O5	-1.08	C6 H10 O5	-1.09	10	2.057	162.053	161.0458	189327	0.117	1378446	0.31
28	Glutaconic acid	C5 H6 O4	-1.85	C5 H6 O4	-1.85	9	2.058	130.0268	129.0196	225246	0.116	1652007	0.38
29	N-Acetylisatin	C10 H7 N O3	-2.98	C10 H7 N O3	-2.98	8	6.791	189.0432	188.0359	336124	0.13	3361253	0.76
30	Ethyl 3-hydroxybutyrate	C6 H12 O3	0.35	C6 H12 O3	0.35	10	7.63	132.0786	131.0714	240477	0.13	2217187	0.50
31	Sinapyl alcohol	C11 H14 O4	0.32	C11 H14 O4	0.32	10	8.43	210.0891	209.0819	546919	0.082	3324441	0.76
32	(R)-Piperazine-2-carboxylic acid			C5 H10 N2 O2	3.15	5	8.62	130.0738	165.0434	6255986	0.104	48668400	11.06
33	2-Dehydro-3-deoxy-D-xylonate	C5 H8 O5	-3.97	C5 H8 O5	-3.97	10	8.62	148.0378	147.0305	217611	0.103	1647012	0.37
34	Ellagic acid	C14 H6 O8	-2.65	C14 H6 O8	-2.65	1	8.994	302.0071	300.9999	1026991	0.095	7621082	1.73
35	Florilenalin	C15 H20 O4	-0.2	C15 H20 O4	-0.2	10	10.054	264.1362	263.1289	250771	0.082	1931176	0.44
36	Geigerin	C15 H20 O4	-1.23	C15 H20 O4	-1.23	10	10.403	264.1365	263.1293	412036	0.078	3231158	0.73
37	Cinncassiol D2 glucoside			C26 H42 O11	2.75	1	10.681	530.2713	565.2405	522384	0.083	3430273	0.78
38	Pyropheophorbide a	C33 H34 N4 O3	3.86	C33 H34 N4 O3	3.86	3	11.114	534.261	533.2531	296611	0.114	5330666	1.21
39	Ligulatin B			C17 H22 O5	-0.62	10	11.123	306.1469	341.1162	463546	0.077	2571120	0.58
40	bipindogenin			C23 H34 O6	-4.08	4	11.306	406.2372	441.2065	710628	0.089	4669690	1.06

**Table 3:** The identified compounds from KH3 extract.

No	Name	MFG Formula	MFG Diff (ppm)	DB Formula	DB Diff (ppm)	Hits (DB)	Retention time (RT)	Mass	Mass-to-charge ratio (m/z)	Height	Width	Volume	Volume (%)
1	2-Chlorobenzhydrol	C13 H11 Cl O	-10.82	C13 H11 Cl O	-10.82	4	0.647	218.0522	217.0435	4097773	0.08	20732970	4.77
2	Nigerose (Sakebiose)	C12 H22 O11	-6.08	C12 H22 O11	-6.08	10	0.649	342.1183	377.0859	4146672	0.1	28923380	6.66
3	Myxothiazol Z			C26 H34 N2 O4 S2	1.38	1	0.672	502.1953	537.1654	686438	0.069	3742336	0.86
4	1,9-Dimethyluric acid	C7 H8 N4 O3	0.22	C7 H8 N4 O3	0.22	7	0.672	196.0596	195.0523	2579719	0.08	12375466	2.85
5	4-Methylene-L-glutamine			C6 H10 N2 O3	-4.4	3	0.682	158.0698	193.0392	144528	0.055	625779	0.14
6	Lathyrine	C7 H10 N4 O2	2.24	C7 H10 N4 O2	2.24	5	0.708	182.08	181.0727	510169	0.122	3135427	0.72
7	1,3,9-Trimethyluric acid	C8 H10 N4 O3	1.53	C8 H10 N4 O3	1.53	10	0.716	210.075	245.0443	211255	0.063	682712	0.16
8	Melizame	C7 H6 N4 O2	-1.13	C7 H6 N4 O2	-1.14	2	0.718	178.0493	177.042	890849	0.064	4304107	0.99
9	Purine	C5 H4 N4	-0.71	C5 H4 N4	-0.71	1	0.72	120.0437	119.0363	123394	0.111	796236	0.18
10	Galactopinitol B	C13 H24 O11	2.49	C13 H24 O11	2.49	4	0.728	356.131	391.1006	422969	0.066	2464180	0.57
11	N-Glycoloyl-neuraminate	C11 H19 N O10	-3.15	C11 H19 N O10	-3.15	3	0.731	325.1019	324.0936	137679	0.058	452954	0.10
12	Mercaptoacetyl-Phe-Leu			C17 H24 N2 O4 S	-4.29	2	0.731	352.1472	387.1144	255484	0.117	2338008	0.54
13	Benazeprilat			C22 H24 N2 O5	0.74	1	0.734	396.1682	431.1376	115282	0.051	493014	0.11
14	(-)-1-Methylpropyl 1-propenyl disulfide	C7 H14 S2	-3.01	C7 H14 S2	-3.01	1	0.735	162.0542	161.0469	297976	0.116	2724779	0.63
15	7,4′-Dihydroxy-5-methoxyflavanone 7-neohesperidoside-4′-glucoside			C34 H44 O19	0.89	5	0.736	756.247	755.2382	199713	0.077	1382106	0.32
16	Glibornuride			C18 H26 N2 O4 S	0	1	0.739	366.1613	401.1306	722659	0.133	4443437	1.02
17	Oxmetidine	C19 H21 N5 O3 S	3.51	C19 H21 N5 O3 S	3.51	7	0.745	399.1351	398.1283	126782	0.078	691340	0.16
18	2(α-D-Mannosyl)-D-glycerate	C9 H16 O9	-2.01	C9 H16 O9	-2.01	6	0.751	268.08	267.0727	228621	0.091	1464340	0.34
19	1-Imidazolelactic acid			C6 H8 N2 O3	-0.42	5	0.751	156.0536	191.023	173275	0.091	891731	0.21
20	3,3-Dimethyl-1,2-dithiolane	C5 H10 S2	-4.79	C5 H10 S2	-4.79	3	0.76	134.023	133.0157	663794	0.075	3217206	0.74
21	Caffeine	C8 H10 N4 O2	0.52	C8 H10 N4 O2	0.52	3	0.767	194.0803	193.073	475137	0.112	7197762	1.66
22	Deoxyinosine	C10 H12 N4 O4	-0.07	C10 H12 N4 O4	-0.07	3	0.777	252.0859	251.0786	518759	0.146	10227982	2.36
23	Glucitol-4-Gucopyanoside	C12 H24 O11	3.32	C12 H24 O11	3.32	6	0.829	344.1307	343.1238	256552	0.072	1088237	0.25
24	Asp His	C10 H14 N4 O5	1.46	C10 H14 N4 O5	1.46	5	0.842	270.096	269.0887	498358	0.149	4407898	1.02
25	10,11-epoxy-chlorovulone I			C21 H29 Cl O5	-4.36	2	0.842	396.1721	431.1399	443250	0.083	2744084	0.63
26	Glu His	C11 H16 N4 O5	2.35	C11 H16 N4 O5	2.35	5	0.851	284.1114	283.1043	1066690	0.084	6256162	1.44
27	Matteucinol 7-O-beta-D-apiofuranosyl(1->6)-beta-D-glucopyranoside	C29 H36 O14	-1.45	C29 H36 O14	-1.45	2	0.858	608.2114	607.2047	292454	0.074	1530638	0.35
28	Benfuracarb			C20 H30 N2 O5 S	3.76	2	0.864	410.186	445.1553	1201767	0.07	6052946	1.39
29	Panose	C18 H32 O16	-1.16	C18 H32 O16	-1.16	10	0.889	504.1696	503.1624	840450	0.077	3427406	0.79
30	Maltopentaose			C30 H52 O26	4.66	4	0.893	828.2708	827.2638	120904	0.034	377181	0.09
31	6-O-beta-D-Xylopyranosyl-D-glucose	C11 H20 O10	-3.62	C11 H20 O10	-3.62	8	0.9	312.1068	311.0995	918890	0.076	3998173	0.92
32	Quercetin 5,7,3′,4′-tetramethyl ether 3-rutinoside	C31 H38 O16	-3.85	C31 H38 O16	-3.85	2	0.9	666.2185	665.2111	161827	0.08	798365	0.18
33	Theobromine	C7 H8 N4 O2	-3.26	C7 H8 N4 O2	-3.26	3	0.904	180.0653	179.0578	471898	0.056	1854968	0.43
34	a-L-Arabinofuranosyl-(1->3)-b-D-xylopyranosyl-(1->4)-D-xylose	C15 H26 O13	0.2	C15 H26 O13	0.2	7	0.908	414.1373	413.13	214008	0.05	816836	0.19
35	2-Propenyl propyl disulfide	C6 H12 S2	-7.16	C6 H12 S2	-7.16	3	0.912	148.0391	147.0315	1589622	0.059	4829204	1.11
36	a-L-Arabinofuranosyl-(1->2)-[a-D-mannopyranosyl-(1->6)]-D-mannose	C17 H30 O15	2.36	C17 H30 O15	2.36	7	0.926	474.1574	473.1503	373430	0.063	1631766	0.38
37	Furcelleran			C31 H27 N O4	3.74	1	0.955	477.1922	512.1619	590749	0.052	2360556	0.54
38	Nω-Acetylhistamine			C7 H11 N3 O	-0.6	3	1.019	153.0903	188.0598	117972	0.053	398648	0.09
39	DL-2-Aminoadipic acid	C6 H11 N O4	-3.64	C6 H11 N O4	-3.64	10	1.402	161.0694	160.0621	129567	0.077	626906	0.14
40	DL-α-Lipoic Acid	C8 H14 O2 S2	-2.24	C8 H14 O2 S2	-2.24	1	1.761	206.044	205.0365	298806	0.065	1436627	0.33
41	Benzocaine	C9 H11 N O2	-1.22	C9 H11 N O2	-1.21	10	1.958	165.0792	164.0725	191008	0.102	1301021	0.30
42	Levoglucosan	C6 H10 O5	-3.47	C6 H10 O5	-3.48	10	2.052	162.0534	161.0462	793853	0.111	6819892	1.57
43	Glutaconic acid	C5 H6 O4	-4.12	C5 H6 O4	-4.12	9	2.053	130.0271	129.0199	864041	0.116	6514179	1.50
44	Flopropione	C9 H10 O4	-0.92	C9 H10 O4	-0.92	10	5.123	182.0581	181.0508	211386	0.261	3881344	0.89
45	Ethyl 3-hydroxybutyrate	C6 H12 O3	-4.28	C6 H12 O3	-4.28	10	7.426	132.0792	131.072	896607	0.124	8466354	1.95
46	PG(19:0/20:1(11Z))			C45 H87 O10 P	-3.57	10	9.437	818.6066	853.576	136863	0.061	857146	0.20
47	Dictyoquinazol C	C18 H18 N2 O5	1.06	C18 H18 N2 O5	1.06	2	9.662	342.1212	341.1139	182205	0.081	1065765	0.25
48	Vardenafil			C23 H32 N6 O4 S	-4.63	1	9.665	488.2228	523.1918	169070	0.07	932207	0.21
49	Cinncassiol D2 glucoside			C26 H42 O11	0.44	1	10.486	530.2725	565.2416	151710	0.054	778059	0.18
50	Gingerol	C17 H26 O4	-0.18	C17 H26 O4	-0.18	7	13.285	294.1832	293.1769	396231	0.094	3300414	0.76
51	Arjunolic acid	C30 H48 O5	-1.49	C30 H48 O5	-1.49	10	13.434	488.3509	487.3447	271061	0.103	3942064	0.91

**Table 4:** Variation in the composition of the detected chemical compounds accross KH1, KH2, and KH3 extracts.

			Source and volume % in each sample		
No	Compound Name	Class of Compound	KH1	KH2	KH3
1	(-)-1-Methylpropyl 1-propenyl disulfide	Organosulfur Compound			+ (0.63)
2	(R)-Piperazine-2-carboxylic acid	Organic Acid		+(11.06)	
3	(S)-Multifidol 2-[apiosyl-(1->6)-glucoside]	Carbohydrate Derivative	+(0.62)		
4	1,10-Phenanthroline	Phenanthrolines	+ (2.56)		
5	1,3,9-Trimethyluric acid	Purines			+ (0.16)
6	1,9-Dimethyluric acid	Purines			+ (2.85)
7	10,11-epoxy-chlorovulone I	Fatty Acid Derivative			+ (0.63)
8	1-Imidazolelactic acid	Carboxylic Acids		+ (0.66)	+ (0.21)
9	1-Methylxanthine	Alkaloids	+ (15.90)		
10	1-Octen-3-ol-3-o-beta-D-xylopyranosyl(1->6)-beta-D-glucopyranoside	Carbohydrate Derivative	+ (0.22)		
11	2(α-D-Mannosyl)-D-glycerate	Glycolipids			+ (0.34)
12	2-Chlorobenzhydrol	Organic Cyclic Compound			+(4.77)
13	2-Dehydro-3-deoxy-D-xylonate	Organic Hydroxy Compound		+(0.37)	
14	2’-Deoxymugineic acid	Carboxylic Acids		+ (0.13)	
15	2-Propenyl propyl disulfide	Sulfur Compounds			+(1.11)
16	3-(3-Methylbutyl)tricetin 5-neohesperidoside	Carbohydrate Derivative	+ (0.19)		
17	3,3-Dimethyl-1,2-dithiolane	Organic Heterocyclic Compound		+ (5.41)	+ (0.74)
18	4,2′,3′,4′-Tetrahydroxychalcone	Flavonoids	+(0.36)		
19	4,4′-Stilbenedicarboxamidine	Benzene Derivatives	+(2.20)		
20	4-Methylene-L-glutamine	Organic Amino Compound		+(1.03)	+ (0.14)
21	5,8,11-Dodecatriynoic acid	Medium-Chain Fatty Acid	+ (0.28)		
22	5-Hydroxy-4-methoxy-3-methyl-2,6-canthinedione	Organic Heterocyclic Compound		+(0.40)	
23	6,7-Dimethyl-9-(2-acetoxyethyl)isoalloxazine	Organic Heterocyclic Compound	+(0.76)		
24	6-Hydroxyl-1,6-dihydropurine ribonucleoside	Glycosides		+ (0.63)	
25	6-O-beta-D-Xylopyranosyl-D-glucose	Oligosaccharide			+(0.92)
26	7,4′-Dihydroxy-5-methoxyflavanone 7-neohesperidoside-4′-glucoside	Carbohydrates And Carbohydrate Derivatives			+(0.32)
27	Adipamide	Amides	+ (0.34)		
28	a-L-Arabinofuranosyl-(1->2)-[a-D-mannopyranosyl-(1->6)]-D-mannose	Oligosaccharide			+ (0.38)
29	a-L-Arabinofuranosyl-(1->3)-b-D-xylopyranosyl-(1->4)-D-xylose	Oligosaccharide			+(0.19)
30	Arabinosylhypoxanthine	Organic Heterocyclic Compound		+(0.35)	
31	Arg His Thr	Amino Acids, Peptides, And Proteins	+(0.55)		
32	Arjunolic acid	Triterpenes			+(0.91)
33	Asp His	Amino Acids, Peptides, And Proteins			+(1.02)
34	Benazeprilat	Heterocyclic Compounds			+(0.11)
35	Benfuracarb	Heterocyclic Compounds			+(1.39)
36	Benzocaine	Carboxylic Acids		+(2.72)	+(0.30)
37	bipindogenin	Cardiac Glycosides		+(1.06)	
38	Caffeine	Alkaloids			+(1.66)
39	Cinncassiol D2 glucoside	Terpenoid	+(0.45)	+(0.78)	+(0.18)
40	Coproporphyrin II	Porphyrins		+(0.66)	
41	D-Ala-D-Ala	Amino Acids, Peptides, And Proteins		+(6.69)	
42	Deoxyinosine	Glycosides			+(2.36)
43	D-α-Hydroxyglutaric acid	Carboxylic Acids	+(0.55)		
44	Dihydroxycarbazepine	Heterocyclic Compounds	+(0.43)		
45	DL-2-Aminoadipic acid	Carboxylic Acids			+(0.14)
46	DL-α-Lipoic Acid	Cyclic Disulfide			+(0.33)
47	Ellagic acid	Heterocyclic Compounds		+(1.73)	
48	Epicatechin 5,3′-dimethyl ether	Flavans, Flavanols And Leucoanthocyanidins		+(0.38)	
49	ethyl 2-cyano-3-(1h-indol-3-yl)prop-2-enoate	Aliphatic Heterocycles	+ (1.17)		
50	Ethyl 3-hydroxybutyrate	Carboxylic Acids	+ (0.43)	+ (0.50)	+(1.95)
51	Flopropione	Polyphenols			+(0.89)
52	Florilenalin	Sesquiterpenoid		+(0.44)	
53	Flumethasone	Corticosteroid		+(0.40)	
54	Fluticasone 17-carboxylic acid	Polycyclic Compounds		+(0.31)	
55	Fluticasone propionate	Steroids		+(0.29)	
56	Furcelleran	Heterocyclic Compounds			+(0.54)
57	Galactopinitol B	Glycosyl Compound			+ (0.57)
58	Geigerin	Sesquiterpenes	+ (3.41)	+ (0.73)	
59	Gingerol	Phenol			+ (0.76)
60	Glibornuride	Sulfonylurea Compounds			+ (1.02)
61	Gln Trp Glu	Organic Amino Compound	+(0.37)		
62	Glu His	Organic Amino Compound			+ (1.44)
63	Glucitol-4-Gucopyanoside	Carbohydrates			+(0.25)
64	Glutaconic acid	Carboxylic Acids		+ (0.38)	+(1.50)
65	HoPhe-HoPhe-OH	Amino Acid Derivatives	+(0.33)		
66	Imetit	Heterocyclic Compounds	+(1.50)		
67	Isobergaptene	Organic Heterocyclic Compound		+(7.29)	
68	Isopropyl apiosylglucoside	Organooxygen Compound		+(0.55)	
69	L-Arginine phosphate	Amino Acids		+(0.32)	
70	Lathyrine	Amino Acids			+(0.72)
71	L-Canaline	Carboxylic Acids	+(0.19)	+(0.25)	
72	Levoglucosan	Organic Compound		+ (0.31)	+(1.57)
73	Lys Phe Cys	Organic Amino Compound	+ (0.68)		
74	Maltopentaose	Organic Compound			+(0.09)
75	Matteucinol 7-O-beta-D-apiofuranosyl(1->6)-beta-D-glucopyranoside	Glycosyl Compound			+ (0.35)
76	Melizame	Organic Compound			+(0.99)
77	Mercaptoacetyl-Phe-Leu	Amino Acids			+ (0.54)
78	Methyl 6-O-digalloyl-beta-D-glucopyranoside	Gallate Ester	+(0.43)		
79	Methylisocitric acid	Organic Hydroxy Compound		+ (0.95)	
80	Metominostrobin	Organonitrogen Compound	+(0.63)		
81	Myxothiazol Z	Thiazoles			+ (0.86)
82	N-(1-Deoxy-1-fructosyl)phenylalanine	Amino Acid Derivative		+ (1.67)	
83	N6-Acetyl-N6-hydroxy-L-lysine	Amino Acids		+(1.62)	
84	N-Acetylisatin	Heterocyclic Compounds		+ (0.76)	
85	N-Desethylquinagolide glucuronide	Quinagolide Derivative	+(0.27)		
86	Neu5Acα2-6GalNAcα-Thr	Amino Acid		+(0.12)	
87	N-Glycoloyl-neuraminate	Ketoaldonate Derivative			+(0.10)
88	Nigerose (Sakebiose)	Ppolysaccharide			+ (6.66)
89	Nω-Acetylhistamine	Organic Compounds			+(0.09)
90	Oxmetidine	Heterocyclic Compounds			+(0.16)
91	Panose	Trisaccharide			+ (0.79)
92	PG(19:0/20:1(11Z))	Lipid			+(0.20)
93	Phe Asn Val	Organic Amino Compound	+(1.00)		
94	Propane-1-thiol	Organic Compound	+(0.44)		
95	Proparacaine	Carboxylic Acids	+(0.30)		
96	Purine	Heterocyclic Compounds			+(0.18)
97	Pyocyanin	Phenazine	+(0.20)		
98	Quercetin 5,7,3′,4′-tetramethyl ether 3-rutinoside	Heterocyclic Compounds			+(0.18)
99	Schizonepetoside C	Organooxygen Compound	+(0.28)		
100	Sinapyl alcohol	Carboxylic Acids		+(0.76)	
101	Strictosamide	Heterocyclic Compounds		+(0.18)	
102	Theobromine	Heterocyclic Compounds		+(1.81)	+ (0.43)
103	Thr-Thr-OH	Organic Amino Compound		+(0.11)	
104	Trimethylolpropane triacrylate	Carboxylic Acids	+(2.58)		
105	Tyrosyl-Methionine	Organic Amino Compound	+(0.36)		
106	Ulexone B	Organic Heterocyclic Compound	+(0.40)		
107	Val Asp	Organic Amino Compound		+(0.44)	
108	Valsartan	Amino Acids	+(0.26)		
109	Vardenafil	Heterocyclic Compounds			+(0.21)
110	Zizybeoside II	Organochalcogen Compound	+ (0.47)		

+indicates presence of compound

## 3 Experimental Design, Materials and Methods

### 3.1 Sample collection and extraction

Three samples of KH were harvested from three locations in Kuantan, Pahang, Malaysia. The stingless bees reared were of the species of *Heterotrigona itama* and *Tetrigona binghami* where the beekeeping were carried out in artificial hive wooden boxes of 37 cm x 37 cm x 8 cm. The beehives of *Heterotrigona itama* were located near to the Bukit Kuin forest (3°52′54.9′′N 103°12′27.4′′E) (designated as KH1), and in front of Kuliyyah of Nursing, International Islamic University Malaysia (IIUM), Indera Mahkota, Kuantan (3°50′28.7′′N 103°18′05.8′′E) (designated as KH2), while the beehive of *Tetrigona binghami* was located at the Sultan Haji Ahmad Shah Mosque, IIUM Kuantan, Indera Mahkota, Kuantan, Pahang (3°50′21.6′′N 103°18′14.3′′E) (KH3). All honey samples were collected on 30^th^ October 2022 during a dry season.

### 3.2 Sugaring-out assisted liquid-liquid extraction (SULLE)

The extraction of compounds from KH was performed according to the SULLE method adopted from Zhu *et al.* [1]. This method is rapid, easy to perform and can provide good extraction efficiency with less consumption of organic solvents and samples. Initially, a total of 0.5 g KH sample was added with 1 mL of acidified water (adjusted the pH to 2.0 using the concentrated hydrochloric acid solution), followed by a vigorous mix until the honey was completely dissolved. Then, 1 mL of acetonitrile was added and vortexed for 1 minute. The mixture was centrifuged (Eppendorf, Germany) at 4000 rpm for 1 minute and the upper organic layer was collected. The chemical extraction using acetonitrile was repeated twice and the collected organic phase was dried in an incubator (TECH-LAB, Malaysia) at 40${\;}^{\circ }C$ overnight. To ensure the complete drying of acetonitrile, the dried extracted samples were further dried under gentle nitrogen flow for 1 hour. The extracts for KH1, KH2, and KH3 were then kept at 4${\;}^{\circ }C$ chiller (Samsung, Malaysia) prior to the subsequent chemical analysis.

### 3.3 Chemical profiling analysis using LC-MS QTOF

The chemical profiling of KH1, KH2, and KH3 was conducted using an Agilent 1290 Infinity LC system coupled to Agilent 6520 Accurate-Mass Q-TOF mass spectrometer with dual ESI source (Agilent Technologies Inc, USA) using the parameters shown in Table 5. Each of the three extracted honey samples was dissolved with 200 µL of methanol/water solution (v/v, 1:1) before being injected into the column. One µL of each extracted sample was injected into an Eclipse XDB-C18 Narrow-bone column (150 mm x 2.1 mm, 3.5-micron, Agilent Technologies Inc, USA). A gradient system consisting of mobile phase A (0.1% formic acid in deionised water) and B (0.1% formic acid in acetonitrile) was used at the flow rate of 0.5 mL/min and column temperature of 25°C. The total runtime was 25 min and the concentration gradient was varied as shown in Table 6.

**Table 5:** The MS operating parameters.

Parameter	Values
Modes	MS only
Ion polarity	Negative
Vcap	3500 V
Fragmentor Voltage	125 V
Skimmer	65 V
OCT 1 RF Vpp	750 V
Drying Gas	10L/min
Gas temperature	300°C
Nebulizer	45 psig
Mass range (m/z)	100 (Min)
	3200 (Max)
Reference ions used	119.03632
	966.000725
Acquisition rate (spectra/s)	1.03
Acquisition time (ms/spectrum)	973
Transient/spectrum	9632

**Table 6:** The gradient system used for the LC-MS/MS-QTOF analysis.

Time (min)	B (%)
0	5
5	5
20	100
25	100

### 3.4 Data acquisition, processing, and reporting

Data was processed with the Agilent MassHunter Qualitative Analysis B.07.00 (Agilent Technologies, United States) software. The raw data of identified compound was processed and the tentative identification of the target compound was performed using the Agilent MassHunter METLIN Metabolomics Database and Library (Metlin_AM_PCDL-N-170502.cdb) by matching mass values with a match tolerance of 5 ppm.

## Ethics Statement

This research work does not require ethical approval.

## CRediT Author Statement

**Ismail C.M.K.H.** Laboratory analysis, writing, original draft preparation; **Khong N.M.H.** Methodology, data curation, reviewing and editing; **Ahmad, A.** Methodology; **Mokhtar K.I.** Conceptualization and methodology; **Lestari W.** Conceptualization, reviewing and editing; Mustafa **Alahmad B.E.** Conceptualization, reviewing and editing. **Abdul Hamid A.A.** Supervision and validation; **Mohd Abd Razak M.R.B.** Supervision and validation; **Ismail A.** Conceptualization, methodology, reviewing and editing. All authors contributed to this manuscript.

## Declaration of Competing Interest

The authors declare that they have no known competing financial interests or personal relationships which have or could be perceived to have influenced the work reported in this article.

## Data Availability

Metabolites in kelulut honey of the stingless bees, Heterotrigona itama and Tetrigona binghami from Kuantan, Pahang, Malaysia: LC-MS/MS-QTOF Dataset (Original data) (Mendeley Data).
